# Platelet-rich plasma enhances the repair capacity of muscle-derived mesenchymal stem cells to large humeral bone defect in rabbits

**DOI:** 10.1038/s41598-020-63496-5

**Published:** 2020-04-21

**Authors:** Nuo Yin, Yifei Wang, Liang Ding, Junjie Yuan, Li Du, Zhongsheng Zhu, Mingmang Pan, Feng Xue, Haijun Xiao

**Affiliations:** Department of orthopedics, Shanghai Fengxian District Central Hospital, Shanghai, 201499 China

**Keywords:** Cell biology, Stem cells

## Abstract

Mesenchymal stem cell-based therapy is a highly attractive strategy that promotes bone tissue regeneration. The aim of the present study was to evaluate the combination effect of muscle-derived mesenchymal stem cells (M-MSCs) and platelet-rich plasma (PRP) on bone repair capacity in rabbits with large humeral bone defect. Precise cylindrical bone defects of 10 mm diameter and 5 mm depth were established in rabbit humeral bones, which were unable to be repaired under natural conditions. The rabbits received treatment with M-MSCs/PRP gel, M-MSCs gel, or PRP gel, or no treatment. The bone tissue regeneration was evaluated at day 0–90 after surgery by HE morphological staining, Lane-Sandhu histopathological scoring, tetracycline detection, Gomori staining and micro-computed tomography. Beyond that, Transwell assay, CCK8 assay, Western blot analysis and ALP activity detection were performed in M-MSCs *in vitro* with or without PRP application to detect the molecular effects of PRP on M-MSCs. We found that the repair effect of M-MSCs group or PRP group was limited and the bone defects were not completely closed at post-operation 90 d. In contrast, M-MSCs/PRP group received obvious filling in the bone defects with a Lane-Sandhu evaluation score of 9. Tetracycline-labeled new bone area in M-MSCs/PRP group and new mineralized bone area were significantly larger than that in other groups. Micro-computed tomography result of M-MSCs/PRP group displayed complete recovery of humeral bone at post-operation 90 d. Further *in vitro* experiment revealed that PRP significantly induced migration, enhanced the growth, and promoted the expression of Cbfa-1 and Coll I in M-MSCs. In conclusion, PRP application significantly enhanced the regeneration capacity of M-MSCs in large bone defect via promoting the migration and proliferation of M-MSCs, and also inducing the osteogenic differentiation.

## Introduction

Large bone defects (LBD) derived from tumor resection surgery or congenital malformation still remains a complicated clinical condition with inadequate repairing even using conventional methods^1,2^. Bone tissue engineering has provided a way of repairing bone defects; autograft, allograft and biomaterials have been applied to fill the defect areas to accelerate osteocyte regeneration^3^. These repair approaches, however, still exhibit apparent limitations, such as the poor histocompatibility of biomaterials^4^ and allograft rejection. Therefore, autologous bone graft still represents the gold standard for bone defect repair, which displays good histocompatibility with almost no immune response^5,6^. In recent decades, instead of direct autologous bone implantation^7^, autologous mesenchymal stem cell (MSC) implantation^8^ and its repair capacity have attracted enormous attention for LBD reconstruction.

Muscle-derived MSCs (M-MSCs)^9^ serve as progenitors of connective tissue cells and are of great importance in adult tissues^10^. M-MSCs exhibit general characteristics of stem cells, and can extensively self-renew and differentiate into diverse cell types, including muscle cells and osteocytes. Unlike bone marrow-derived MSCs (BM-MSCs)^11^, M-MSCs can be easily obtained from adult muscle tissues, which accounts for about one third of body weight. M-MSCs are easy to be separated from muscles and cultured *in vitro* as well. These characteristics lay a solid foundation for its role as seed cells in bone repair^12^. M-MSC implantation displays incomparable advantages for bone regeneration, especially for reconstruction of periosteum-damaged bone defects^13,14^. In addition, latest research has proved that M-MSCs can not only directly participate in the repairing of bone defects, but also promote the secretion of paracrine factors to regulate repair process, which is similar to BM-MSCs^15^. Thus, it is tempting to make further exploration on whether M-MSCs can promote the repair of LBDs.

In bone tissue engineering, bone repair not only depends on the seed cells, but also on the extracellular cytokines. In recent years, the application of platelet-rich plasma (PRP) capable of releasing and storing cytokines in tissue engineering becomes more and more popular^16^. As reported, the platelet concentration of PRP is much higher than the basic level in the blood. PRP are also rich in various active factors, such as transforming growth factor and fibrinogen^17^. Additionally, the proportion of each growth factor is consistent with the normal ratio in the body, which is the best synergy between various growth factors. It compensates for the shortcomings of wound repair involving a single growth factor. However, there are scarce reports on whether PRP is able to promote large bone repair.

PRP is also reported capable of inducing the proliferation and migration of periodontal cells^18^. It indicates that PRP may promote the proliferation and migration of M-MSCs to accelerate the bone repair process. However, it is still unclear whether PRP induced the osteogenic differentiation of M-MSCs and whether PRP application could enhance the regeneration capacity of M-MSCs in LBD. In this study, we utilized precise drilling machine to make LBD in rabbit humeral bone. Results proved that M-MSCs/PRP combined therapy significantly promoted the morphological and histological repair of bone defects as compared with M-MSCs or PRP monotherapy. Additionally, M-MSCs/PRP induced the generation of new bone and recovered bone defect at post-operation 90 d via imageological examination. PRP promoted the migration and proliferation, increased the expression of Cbfa-1 and Coll I, and enhanced ALP activity in cultured M-MSCs. It underlies the molecular mechanism of M-MSCs/PRP combined therapy in promoting the repair of LBD. This study provides evidence for the clinical application of M-MSCs/PRP combination and presents a new efficient approach to promote LBD repair.

## Material and Methods

### Animals

Sixty 8-month-old male New Zealand White rabbits weighing 2.5 kg to 3.5 kg were purchased from the Experimental Animal Center of Shanghai Fengxian District Central Hospital. The rabbits were raised individually in standard cages under controlled temperature (25 °C) and light condition, with free access to chow diet and water *ad libitum*. The overall status was monitored every day during pre-operation time, and twice a day during post-operation time. As to allocation, 3 rabbits were randomly chosen for *in vitro* experiments, 14 for blank control, receiving no treatment, 14 for M-MSCs single treatment, 14 for PRP single treatment, and 15 for M-MSCs/PRP combined treatment. The rabbits were excluded for the following experiments once experiencing any serious adverse events like infection, and weight loss. In total, 2 rabbits from blank control group, 2 from M-MSCs group, 2 from PRP group, and 3 from M-MSCs/PRP group, were excluded from this study. There were 12 rabbits left for each group, and 3 rabbits for *in vitro* experiments. Bone repair was examined by micro-computed tomography (micro-CT) at four post-operation time points, 0 d, 30 d, 60 d, and 90 d, before sacrificing. The humeral tissues were removed out and fixed for following experiments. This study was approved by the Animal Ethics Committee of Shanghai Fengxian District Central Hospital, and the rabbits were treated according to the national principles of laboratory animal care and management.

### M-MSCs culturing *in vitro*

The left gluteus maximus of each included New Zealand White rabbit was dissected under aseptic condition. Muscle tissues were then cut into small pieces (1–2 mm^3^), followed by thorough washing with in Ca^2+^/Mg^2+^ free phosphate buffered saline for removing blood cells. The muscle tissues were dried on the clean bench and then placed in the sterile petri dish containing 1% collagenase for digestion. The digested tissues were stirred on a stirrer for 1 h at 37 °C. After centrifugation, 5 ml Dulbecco’s Modified Eagle Medium (DMEM) was added to resuspend cells. Cells were repeatedly pipetted several times, and filtered through a fine (100/i) mesh wire screen to obtain a coarse single-cell suspension. A gradient cell separation medium (Sigma) at a density of 1.088 g/ml was used for lymphocyte separation. After washing, the viable cells were counted to adjust the cell concentration. The cells were then seeded in gelatin-coated 6-well plates at a density of (1–2) * 10^5^/cm^2^ and 3 ml per well, and cultured in DMEM/F12 supplemented with 10% fetal bovine serum (FBS; Hyclone, USA) and 10 ng/ml basic fibroblast growth factor (Sigma). The cells were incubated for 2 h at 37 °C with 5% CO_2_ and saturated humidity. After that, all non-adherent cells were gently transferred from the liquid to another petri dish. The adherent cells were then cultured, and medium were replaced at 3-day intervals. For *in vitro* experiments, 15 mm glass slides were inserted into each well of a 24-well plate for transferring M-MSCs, and then cell fixation was performed using absolute methanol solution.

### Preparation of autologous PRP

Approximately 10 ml rabbit blood was aspirated from the ear central artery into the test tube containing sodium citrate for each New Zealand White rabbit one month before operation. Thereafter, blood collection was performed at one-week intervals, and about 5 ml blood was obtained each time. Autologous PRP was prepared by two-step centrifugation according to the Landsberg’s method^19^. Briefly, initial centrifugation was performed at 400 g for 10 min to remove partial red blood cells and the upper serum, and the centrifuged mixture was re-centrifuged at 1700 g for 10 min to remove all serum and red blood cells, whereby PRP was obtained. Platelet examination showed that the platelet count in PRP was approximate 1527 * 10^3^/μl, which was much higher than that (near 250 * 10^3^/μl) in rabbit blood.

### Preparation of M-MSCs/PRP alginate gel

Alginate gels can function as carriers capable of delivering drugs to defect area, and in the meantime provide a 3-dimensional environment that helps in maintaining aspherical osteoblast morphology of the encapsulated MSCs. Preparation of alginate gels was performed according to Kamarul’s protocol^20^. In brief, rabbit autologous M-MSCs was separated from petri dish surface via pancreatic enzymes. M-MSCs were then centrifuged and resuspended by alginate solution to the concentration of about 1.5 * 10^6^ cells/ml. Subsequently, the alginate solution was transferred to 102 mM CaCl_2_ aqueous solution for solidification, and 10 min later, alginate gels were formed, followed by washing using 0.15 M NaCl aqueous solution. For preparation of M-MSCs/PRP gel, centrifuged M-MSCs were resuspended by PRP (containing alginate) to make a mixed gel of M-MSCs and PRP. For preparation of PRP gel, only PRP (containing alginate) solution was transferred to 102 mM CaCl_2_ aqueous solution for solidification. The three kinds of gels were finally transferred to clean petri dishes under aseptic condition for later filling in bone defect areas. Additionally, to avoid immune response, autologous PRP blood sample was prepared one month before operation for each experiment rabbit. Autologous M-MSCs was obtained from gluteus maximus and cultured two weeks before operation. Thus, both M-MSCs and PRP belongs to complete autografts.

### Construction of humeral bone defects in rabbits

**Figure 1:**
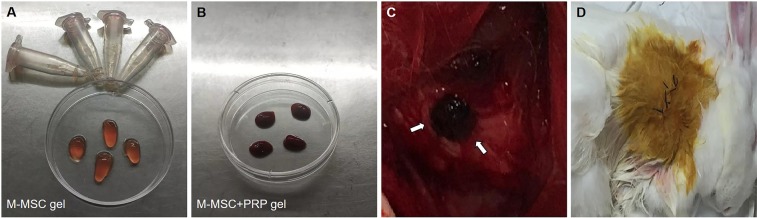
Construction of large bone defect in rabbit humeral bone. (**A**) Exposure of the greater tuberosity of humeral bone. (**B**) The precise drilling device was used to make almost the same bone defects among rabbits. (**C**) The diameter of a defect was 10 mm, and the depth was 5 mm. (**D**) The overall situation of left humeral bone was examined by micro-computed tomography 3D imaging.

Fifty-seven New Zealand White rabbits were given general anesthesia with 1 ml of 2% sodium pentobarbital per kg body weight. Half of the total amount of sodium pentobarbital solution was injected rapidly into the ear vein, and the rest was administered with intravenous infusion in a low-flow rate until the end of operation. After complete anesthesia, hair removal and decontamination were performed on the right upper limb. In order to reach almost the same bone defect among fifty-seven rabbits, both the right upper limb and the automatic drilling device were accurately fixed on the operating table. After fixation, a small incision about 2.5 cm was made on the outside of the proximal humeral bone. Then the muscle tissues were separated layer by layer until the proximal side of the proximal humeral bone was fully exposed (Fig. 1A). The greater tuberosity of humeral bone was exposed in sight. Then cylindrical bone defect of 10 mm diameter and 5 mm depth was established in the lateral side of the greater tuberosity of humeral bone (Fig. 1C) by using the precise drilling device (Fig. 1B). After construction of bone defect, the overall situation of humeral bone was examined by a Biograph 3D micro-CT device (ZKKS-MCT-Sharp, Guangzhou Zhongke Co., China; Fig. 1D). After the operation, broad-spectrum antibiotics were administered among all rabbits for 3 d. The right limbs of rabbits were bandaged and fixed as well. During experiment period, right upper limbs remained immobilized to avoid stress on the bone defects. Once receiving serious complications like infection and weight loss, rabbits would be excluded from the following experiments.

### Implantation of M-MSCs or PRP alginate gels into bone defects

**Figure 2:**
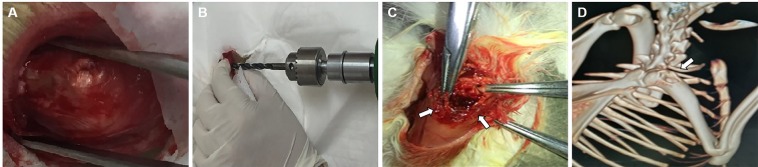
Implantation of autologous M-MSCs or PRP alginate gels into bone defects. (**A**) Preparation of M-MSCs gel. (**B**) Preparation of M-MSCs/PRP gel. (**C**) The defect area was covered with M-MSCs/PRP gel. (**D**) The skin layer was sutured and decontaminated at the end of operation.

After well construction of bone defects of the left humeral bones, the rabbits were randomly divided in four groups. The blank control group did not receive any treatment and defect repair was performed under natural condition. In M-MSCs group, M-MSCs alginate gels (Fig. 2A) were placed on the surface of a defect area, and the gels were covered by muscle flap, followed by closing with absorbable suture. The skin layer was sutured and decontaminated at last (Fig. 2D). For PRP group, the implantation of M-MSCs alginate gels into a defect area was replaced by PRP alginate gels, and other operations or treatments were the same as M-MSCs group. Likewise, M-MSCs/PRP alginate gels were used in M-MSCs/PRP group (Fig. 2B,C). After those operations, the rabbits were raised in cages and observed in the following 90 d.

### Micro-CT detection

The overall defect situation of each rabbit in the four groups was evaluated by micro-CT 3D imaging examination. Defect repair condition was observed at four post-operation time points, 0 d, 30 d, 60 d, and 90 d, by the Biograph 3D micro-CT device (ZKKS-MCT-Sharp, Guangzhou Zhongke Co., China), and the parameters of new bone volume and bone mineral density (BMD) were analyzed by ZKKS-Micro-CT 4.1 software. After micro-CT detection, rabbits were sacrificed at the aforementioned four time points. General anesthesia was performed in the rabbits with 2% sodium pentobarbital. Then cylindrical bone repair tissues of 30 mm diameter and 10 mm depth were obtained.

### Tissue fixation and hematoxylin and eosin (HE) staining

After collection, samples were fixed with 4% paraformaldehyde solution, decalcified in 10% formic acid solution, and embedded in paraffin. The paraffined tissues were then sliced into 10-μm thick sections, and the sections were mounted on glass slides for drying.

For HE staining, paraffin was solubilized at high temperature. Sections were then dewaxed with xylene, dyed with hematoxylin for 2–5 min, rinsed with running water for 5–10 min, and immersed in 1% hydrochloric acid solution for 2–3 s. After that, the sections were dyed with eosin for 1 min. Subsequently, the sections were rinsed with running water for 3 min, dehydrated with gradient alcohol solution, immersed in xylene, and sealed with neutral resin.

### Immunocytochemistry staining

M-MSCs slides were fixed in absolute methanol solution, and then blocked with 5% BSA solution at 37 °C for 1 h. The slides were incubated overnight at 4 °C with primary antibodies: mouse anti-CD 90 antibody (1: 200; Abcam), mouse anti-CD 105 antibody (1: 200; Abcam), and mouse anti-CD 45 antibody (1: 200; Abcam). On the next day, the slides were incubated with secondary antibodies: anti-mouse FITC (1: 200). Final incubation of the slides with DAPI (1: 1000) was performed before sealing with fluorescent gel.

### Tetracycline detection

On the 13th or 14th day before sacrificing, 25 mg/kg (0.1 ml/100 g) of tetracycline hydrochloride was injected subcutaneously into the rabbits. On the third or 4th day before sacrificing, 5 mg/kg (0.1 ml/100 g) of 0.5% calcein was injected subcutaneously into the rabbits. Ten-day interval between the two injections was guaranteed. The bone tissues were then obtained at 30 d, and 90 d after surgery. Tetracycline signal was detected by fluorescence imaging and new bone formation was marked as green fluorescence signal.

### Radiological evaluation

For histological evaluation, bone repair was evaluated by two independent observers (Nuo Y and Yifei W) using Lane-Sandhu histopathological scoring system (Supplementary Table 1). The average value of two measurements was then calculated for statistical analysis.

### Gomori staining

Briefly, paraffin sections were dewaxed by conventional method and stained with lapis lazuli blue dye and alum hematoxylin dye. After that, hydrochloric acid alcohol was used to wash sections until the nuclei turned blue and the background were colorless. The sections were then stained with chromotrope 2 R dye for 10 min and washed by 0.2% acetic acid solution. The mineralized bone presented in red while the other parts were colorless. After being re-stained with brilliant green dye solution, the sections were sealed for microscopy observation.

### CCK8 assay

For detecting cell viability, M-MSCs in the logarithmic growth phase were collected. After cell counting on the blood cell count plate, M-MSCs were added to a 96-well plate, 5000 cells per well. Three replicate wells were set for each group. DMEM was then added to a final volume of 200 μl. The cells were incubated at 37 °C with 5% CO_2_ for 1 d, 2 d, 3 d, 4 d, 5 d, 6 d, and 7 d. Before reading the optical density values, 20 μl CCK8 solution was added to each well and the incubation was continued for 2 h at 37 °C in a 5% CO_2_ incubator. After that, the absorbance was measured at 450 nm using a microplate reader. The growth curve was drawn according to the absorbance. For PRP treatment, M-MSCs were pre-treated with PRP for 24 h before the CCK8 assay.

### Transwell assay

About 80 μL Matrigel was evenly spread on the Transwell chamber and incubated for 2 h at 37 °C. Cells of each group were prepared as cell suspension, and the cell density was adjusted to 2 * 10^5^ cells per ml. A total of 200 μL suspension was transferred to the chamber, and then DMEM medium supplemented with 10% FBS was added into the lower chamber. After incubation for 24 h at 37 °C, the cells in the upper chamber were wiped out with a wet cotton swab, and the chamber was fixed with absolute methanol solution and stained with crystal violet dye. For PRP group, DMEM supplemented with 10% FBS was replaced by PRP. The numbers of stained cells in 10 randomized fields of view per assay were recorded for cell migration analysis.

### Western blot analysis

Cultured cells were harvested by RIPA buffer. The total protein samples were quantified by BCA protein assay and layered by sodium dodecyl sulfate polyacrylamide gel electrophoresis. After that, the proteins were transferred to polyvinylidene fluoride membranes, and the membranes were then blocked by PBST buffer containing 5% bovine serum albumin for 1 h. Subsequently, the membranes were incubated with primary antibodies (1: 500–1000) at 4 °C for more than 12 h, followed by washing with PBST buffer and incubation with secondary horseradish peroxidase-conjugated antibodies at 37 °C for 3 h. At last, the membranes were exposed to an X-ray film in the dark and protein bands were obtained by chemiluminescence imaging. The gray values of the bands were calculated using ImageJ software.

### Alkaline phosphatase activity detection

This detection was performed by Alkaline Phosphatase Activity (ALP) Colorimetric Assay Kit (BioVision, USA). In brief, cultured cells were suspended in 50 ul Assay Buffer and centrifuged at 13000 rpm for 3 min. After that, 20 ul sample was extracted, followed by adding Assay Buffer to achieve a volume of 80 ul for 6 replicates in a 96-well plate. 50 ul of 5 mM p-nitrophenyl phosphate solution was added into each replicate and reacted with the sample at 25 °C for 60 min. The reaction was then stopped by 20 ul termination solution. Absorption value of each reaction mixture was measured at 405 nm wavelength.

### Statistical analysis

The positive signal area, the new bone area or the number of migrated cells in the graph were statistically analyzed using Image Pro Plus software. The statistical data were presented as mean ± standard deviation ($\bar{x}$ ± SD). The data between two groups were compared using independent-samples t-test. The data between groups were compared by one-way analysis of variance. For all analyses, P values less than 0.05 were considered statistically significant.

## Results

### *In vitro* identification of M-MSCs

**Figure 3:**
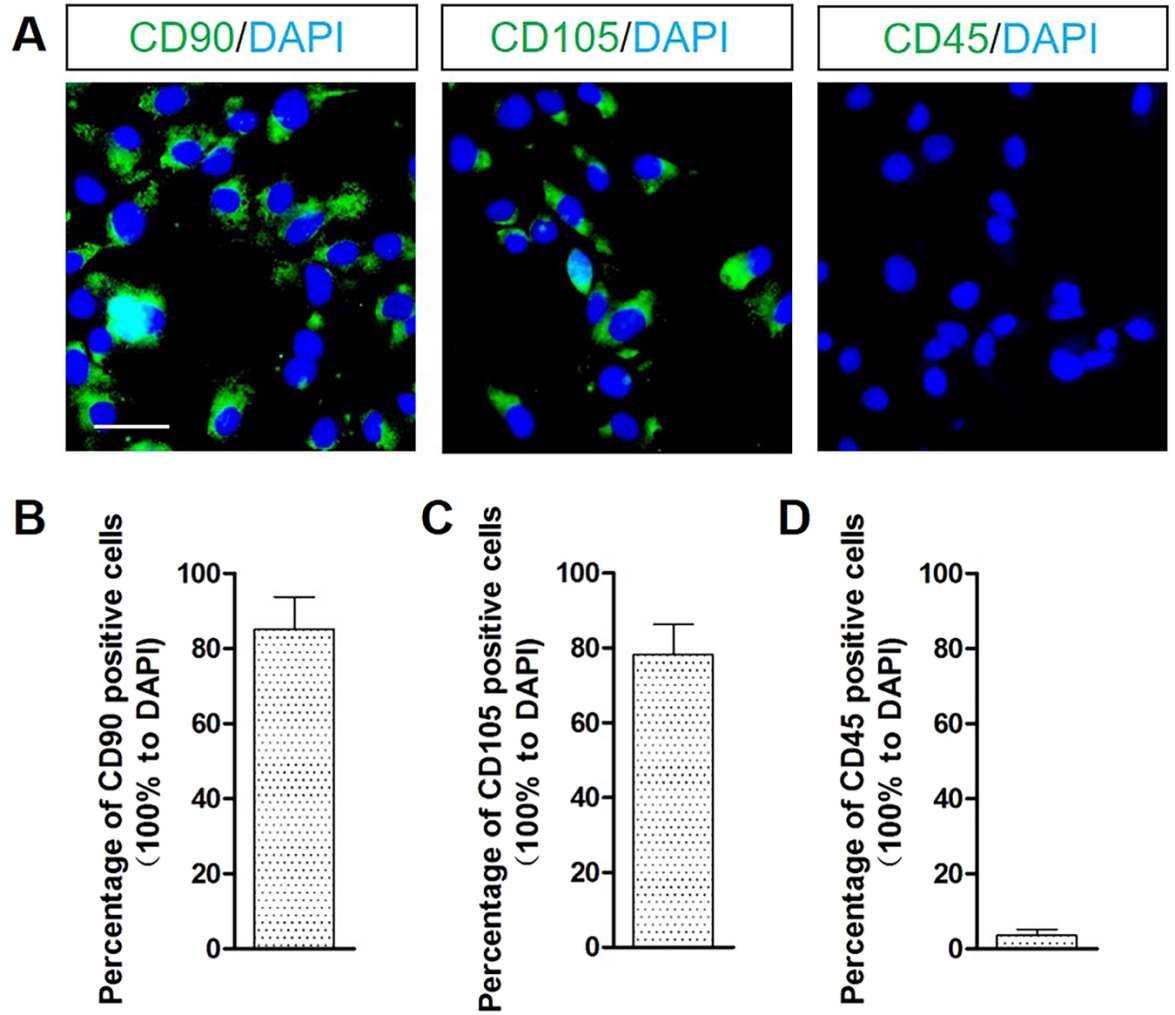
*In vitro* identification of M-MSCs. (**A**) CD 90 and CD 105 fluorescence signals were positive and CD 45 fluorescence signal was barely positive among cells. Scale bar = 20 μm. (**B**) Percentage of CD 90 positive cells was 85%. (**C**) Percentage of CD 105 positive cells was 78%. (**D**) Percentage of CD 45 positive cells was below 5%. 10 randomized fields of view for each group.

To verify whether M-MSCs have been successfully cultured *in vitro*, we firstly performed immunocytochemistry staining to detect the crucial biomarkers in the cells. Results showed that CD 90 and CD 105 were positive among cells, and the positive rates for CD 90 and CD 105 were 85% and 78%, respectively (Fig. 3A,B). These strongly suggest that the cells were MSCs and the purity was high. However, CD 45 fluorescence signal was barely positive among cells (Fig. 3C), with a positive rate of below 5%. It provided evidence that these stem-like cells were not derived from the flowing hematopoietic cells but from muscle cells.

### M-MSCs/PRP gel significantly improved morphological and histological repair in bone defects

**Figure 4:**
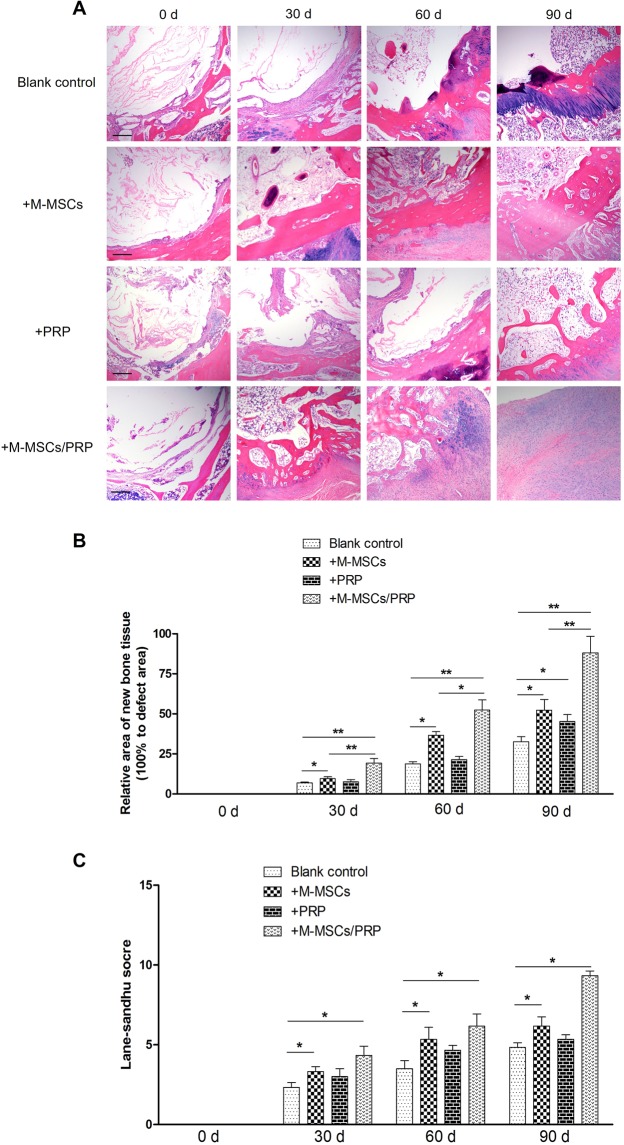
M-MSCs/PRP gel significantly improved morphological repair in bone defects. (**A,B**) HE staining results displayed that compared with blank control group, M-MSCs treatment improved morphological repair in defect area; PRP treatment induced the growth of new bone tissues towards the central of a defect; M-MSCs/PRP treatment significantly promoted the growth of new bone tissues and morphological repair of defects. Compared with blank control group, the repaired area was significantly increased in both M-MSC group and M-MSCs/PRP group. But M-MSCs/PRP group presented significantly higher increase than M-MSC group. 3 randomized fields of view for each group. Scale bar = 250 μm. (**C**) Lane-Sandhu histological evaluation showed that compared with blank control group, significant improvement existed in M-MSCs and M-MSCs/PRP groups at 30 d, 60 d, and 90 d. However, the improvement extent of M-MSCs/PRP group was the largest, which was apparently higher than M-MSCs single treatment. 3 randomized fields of view for each group, *P < 0.05, **P < 0.01.

After humeral bone defect was successfully established in rabbit model, M-MSCs and PRP were utilized individually or jointly to induce defect repair. We firstly performed HE staining to evaluate the morphology alterations of bone defects among groups. As shown in Fig. 4, HE staining results in blank control group displayed that the morphological repair was slow and unbalanced. At 30 d, a thin layer of fibrous tissues formed on the surface of defect area, and chondrocytes induced osteogenesis at 60 d. However, the new bone tissues were not smooth and small defects still scattered in several regions. Even at 90 d, the repair area was still limited and the big defect was not fully filled. In M-MSCs group, fibrous tissue repair was fast and fibrous layer was thick at 30 d. Then osteogenesis was initiated towards the central of defect with time prolonged. However, M-MSCs single treatment also failed to fully fill the defect area after 90 d. In PRP group, fibrous tissue repair and osteogenesis were slow as well, similar to the blank control group. But PRP single treatment induced the early growth of fibrous tissues and new bone tissues towards the central of defect. In M-MSCs/PRP group, fibrous tissue repair and osteogenesis were significantly improved. The new bone tissues morphologically presented lamellar appearance with an obvious growth towards the central of defect even at 30 d. At 60 d, the defect area was surrounded by new bone tissues and its repairing area began to fuse. At 90 d, the whole defect area was filled with new cells, and new bone was formed. Bone defect was morphologically repaired with the combined treatment of M-MSCs/PRP.

Compared with the blank control group, the repair area was not increased significantly in PRP group at 30 d and 60 d. But at 90 d, a significant difference was found between these two groups (Fig. 4B, P < 0.05). It indicates that PRP single treatment could not efficiently and rapidly promote defect repair in rabbit model. Additionally, the repair area was significantly increased in both M-MSC group and M-MSCs/PRP group as compared with the blank control group (both P < 0.05). But the increase of M-MSCs/PRP group was significantly higher than that of M-MSC group (P < 0.05). It indicates that M-MSCs single treatment efficiently induced new bone growth, and this phenotype was amplified with the combination of M-MSCs/PRP. M-MSCs were conferred with an enhanced repair capacity with the help of PRP.

Histological assessment was performed by two independent observers according to Lane-Sandhu histological evaluation scale and the average values were calculated for statistical analysis. Results showed that significant improvements were achieved in M-MSCs, PRP, M-MSCs/PRP groups at 30 d, 60 d, and 90 d as compared with the blank control group (Fig. 4C, all P < 0.05). However, the improvement extent of M-MSCs/PRP group was the largest, which was also apparently higher than that of M-MSCs group. It suggests that M-MSCs/PRP combined treatment accelerates the histological repair process of bone defects.

### M-MSCs/PRP gel significantly improved the formation of new bone tissues

**Figure 5:**
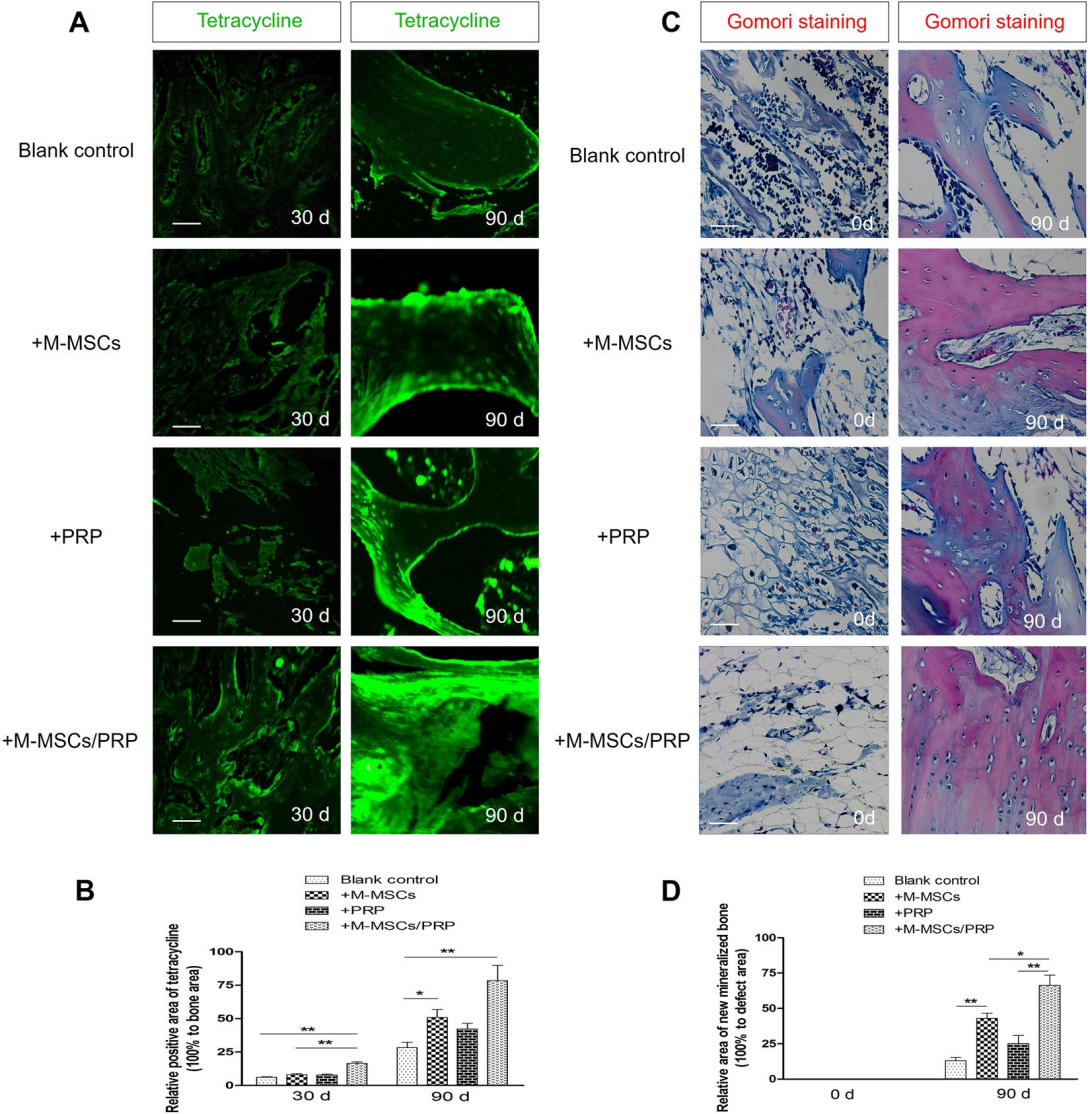
M-MSCs/PRP gel significantly improved the formation of new bone tissues. **(A,B)** Compared with blank control group, tetracycline fluorescence signal was not significantly enhanced in PRP group until 90 d; both M-MSCs and M-MSCs/PRP group displayed apparent tetracycline fluorescence signal. Compared with M-MSCs single group, M-MSCs/PRP group displayed stronger tetracycline fluorescence signal. Scale bar = 100 μm. **(C,D)**, The new mineralized bone was labeled “red” by Gomori staining. Compared with blank control group, M-MSCs group or PRP group, the new mineralized bone area was significantly increased in M-MSCs/PRP group at 90 d. Scale bar = 100 μm. 3 randomized fields of view for each group, *P < 0.05, **P < 0.01.

To further explore the formation of new bones among groups, we utilized tetracycline to mark new bones and Gomori staining to reveal the new mineralized bone. Results showed that compared with the blank control group, tetracycline fluorescence signal was not significantly enhanced in PRP group until 90 d (Fig. 5A, P < 0.05), while both M-MSCs and M-MSCs/PRP groups displayed apparent tetracycline fluorescence signal (both P < 0.05). Additionally, M-MSCs/PRP group displayed stronger tetracycline fluorescence signal as compared with M-MSCs group (P < 0.05).

Statistical analysis also confirmed that the positive areas of tetracycline fluorescence were significantly increased in M-MSCs, PRP and M-MSCs/PRP groups at 90 d as compared with the blank control group (Fig. 5B, all P < 0.05). The positive area of tetracycline fluorescence even reached approximately 80% in M-MSCs/PRP group, which was the strongest improvement among groups.

In addition, the Gomori staining provided histological evidence of new formation of mineralized bone. As show in Fig. 5, the newly formed mineralized bones were dyed red, and the area of now mineralized bones in M-MSCs/PRP group was significantly higher than that in either M-MSCs or PRP group (Fig. 5C, D, P < 0.05).

### M-MSCs/PRP gel recovered bone defects in imageological examination

**Figure 6:**
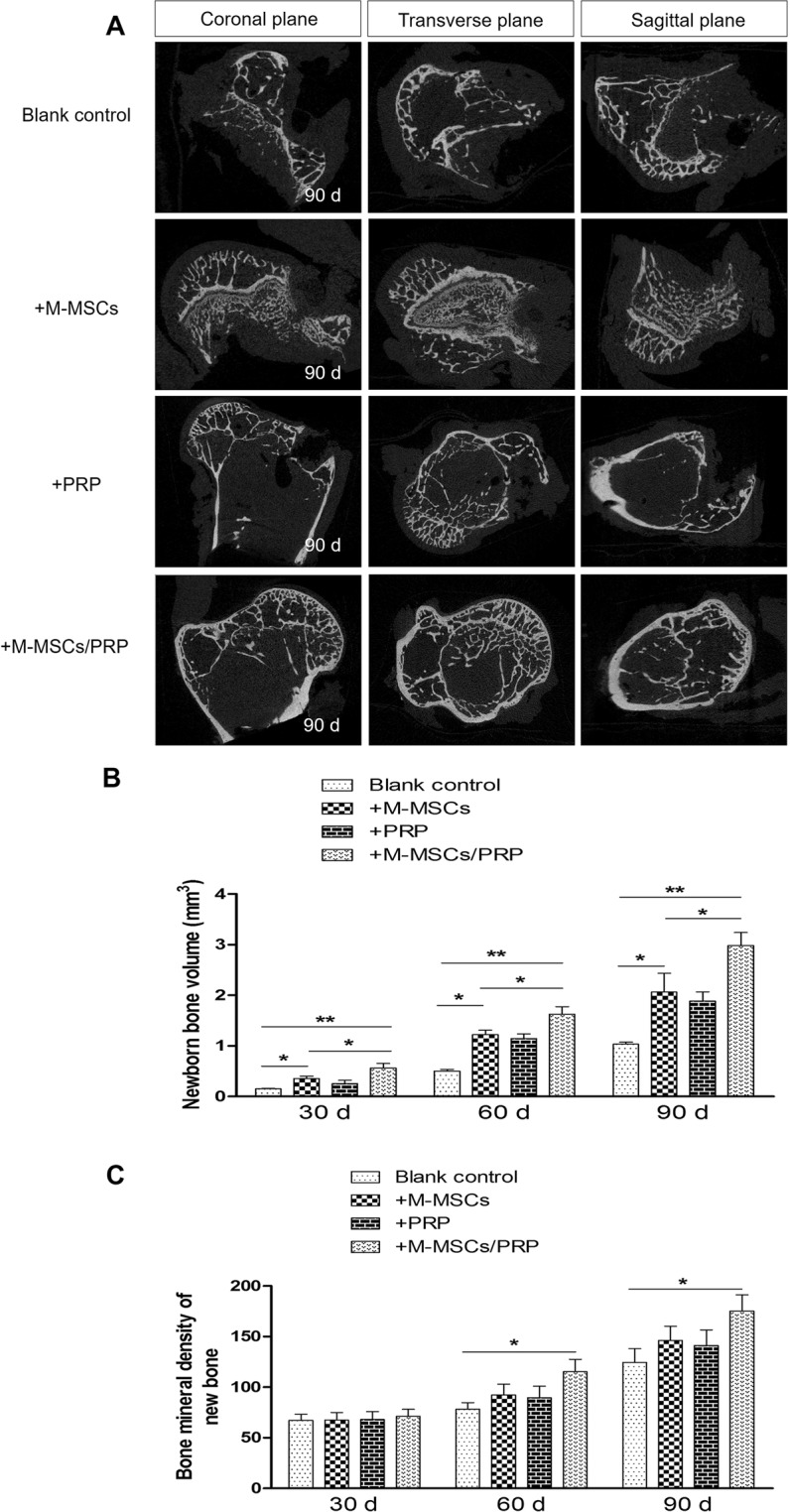
M-MSCs/PRP gel recovered bone defects in imageological examination. (**A**) Micro-computed tomography results displayed that even after 90 d, bone defects were still not closed and the gaps were not filled with new bone tissues in blank control group, which was similar to PRP group. In M-MSCs group, the bone defects were partially closed, but few new bone tissues were observed. In M-MSCs/PRP group, the bone defects were completely closed and the gaps were completely filled with new bone tissues. (**B**) Bar graph of new bone volume at different time points. (**C**) Bar graph of bone mineral density of new bone tissues at different time points. 3 randomized fields of view for each group, *P < 0.05, **P < 0.01.

To verify whether the formation of new bones in M-MSCs/PRP group recovered bone defects, we utilized micro-CT to detect the recovery condition among groups, which was the gold standard in traumatology. Micro-CT result showed that even after, bone defects were still not closed and the gaps were not filled with now bone tissues in the blank control group, which was similar to PRP group. Despite the bone defects were partially closed in M-MSCs group, few new bone tissues were observed. In M-MSCs/PRP group, the bone defects were completely closed, new bone tissues had filled the gaps, and cancellous bones were remodeled at 90 d, which could be diagnosed as imageological recovery (Fig. 6A).

Additionally, we detected new bone volume and BMD at different time points via micro-CT examination. Results showed that new bone volume in either M-MSCs, PRP or M-MSCs/PRP group was significantly larger than that in the blank control group. However, the alteration extent in M-MSCs/PRP group was the largest, which was significantly higher than that in M-MSCs group (Fig. 6B, P < 0.05). BMD was elevated in M-MSCs and PRP groups, but with no significant difference as compared with the blank control group. Significant elevation only existed in M-MSCs/PRP group (Fig. 6C, P < 0.05). It indicates that M-MSCs/PRP combined treatment promotes the enhancement of BMD and the growth of new bone, thus resulting in the imageological recovery of bone defect.

### PRP treatment promoted migration, proliferation and induced osteogenic differentiation of M-MSCs *in vitro*

To further detect the mechanism of M-MSCs/PRP combined therapy in promoting bone repair, we treated M-MSCs with PRP *in vitro* and detected the cellular migration, proliferation and differentiation direction.

**Figure 7:**
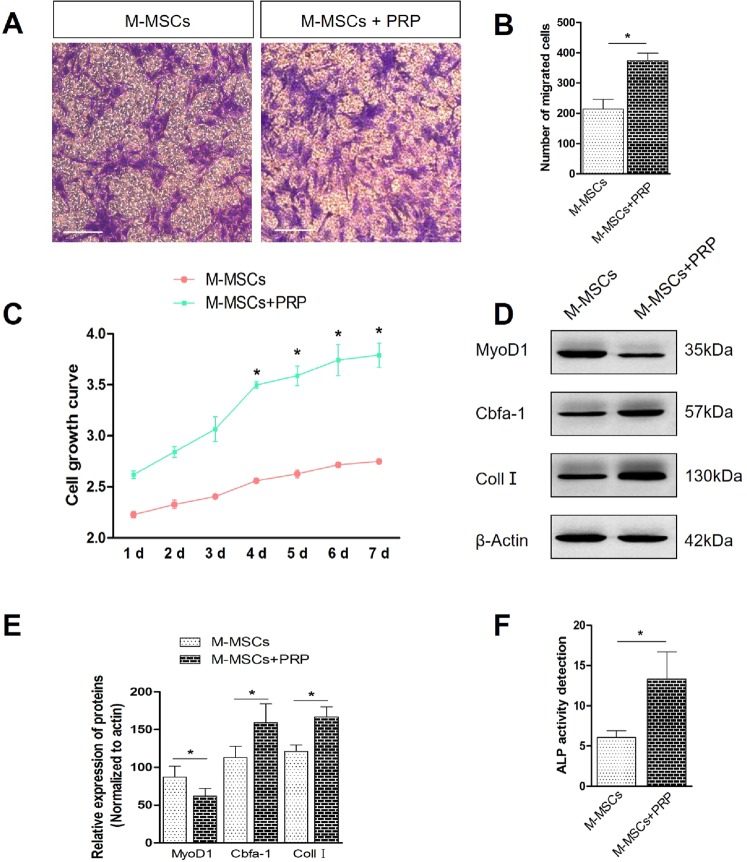
PRP treatment promotes migration, proliferation and induced osteogenic differentiation of M-MSCs *in vitro*. (**A,B**) Transwell assay displayed that after PRP treatment, the number of migrated M-MSCs was significantly increased. 10 randomized fields of view for each group. scale bar = 20 μm. (**C**) CCK8 assay displayed that after PRP treatment, cellular growth was significantly enhanced. (**D,E**) Western blot analysis displayed compared with M-MSCs treated with FBS, PRP treatment increased the expression of Cbfa-1 and Coll I while decreased the expression of MyoD1. (**F**) ALP activity detection revealed PRP treatment significantly elevated ALP activity in differentiated M-MSCs. *P < 0.05, **P < 0.01.

Transwell assay was performed to detect the migration of M-MSCs *in vitro*. Results showed that after PRP treatment, the number of migrated M-MSCs was significantly increased (Fig. 7A, B, P < 0.05), compared with M-MSCs treated with FBS. CCK8 assay also showed the cellular growth curve was significantly different since day 4. PRP treatment significantly increased the proliferation of M-MSCs, compared with M-MSCs treated without PRP (Fig. 7D, P < 0.05). Then, we performed western blot analysis to detect the differentiation direction of M-MSCs under PRP treatment. Result displayed the transcription factor for muscular differentiation, MyoD1 was significantly decreased while the crucial transcription factor for osteogenic differentiation, Cbfa-1 was significantly increased, which was also accompanied with the elevation of Coll I expression after PRP treatment (Fig. 7E, F, P < 0.05). Finally, the osteogenic differentiation of M-MSCs *in vitro* was also confirmed by ALP activity detection. Result displayed a multi-fold increase of ALP activity of differentiated M-MSCs after PRP treatment, compared with M-MSCs treated with FBS. Taken together, these results indicate PRP have strong motivative effects on M-MSCs. It enhances the migration, proliferation capacity of M-MSCs and also induces the osteogenic differentiation of M-MSCs, which underlies the strong regeneration capacity of M-MSCs/PRP combined therapy in LBD.

## Discussion

LBDs arising from tumor resection are not always healed by the intrinsic regenerative capacity of the bone. That means spontaneous bone regeneration in LBDs in morphology cannot be well achieved without the aid of any certain treatment. Thus, the LBD model better reflects the osteogenic potential and wound repair capacity of bone engineering graft. Studies have reported that the critical size of LBD should be 1.5–2.5 times the bone diameter or one-tenth of the bone length^21^. The average length of humeral bones of New Zealand White rabbits was 8 cm. Therefore, to construct LBD rabbit model, the defect diameter should be more than 8 mm. In our experiment, cylindrical LBD of 10 mm diameter and 5 mm depth were established in rabbit humeral bones using a precise drilling device. In blank control group, at 90 d after bone defect construction, HE stanning was performed for morphological evaluation. The repaired area was only 32% of the total defect area. Bone defect area was not fully filled and only a surface layer formed on the defect area. The new bone tissues were not smooth and small defects still existed. Additionally, tetracycline-labeled new bone area was visually less than 25% of the total bone defect area and new mineralized bone area was even less. Histological evaluation showed that even after 90 d of natural bone repair with the endogenous healing capacity, the Lane-Sandhu score remained at 4.5, which displayed an impaired bone repair process with poor repair effect. Micro-CT results showed that LBD of blank control group were still existed, defect areas were not closed and BMD was low. These results indicate that the LBDs in humeral bones of New Zealand White rabbits were successfully modeled. Without treatment intervention, even after 90 d, the bone defects cannot be naturally closed, and the natural repair effect is poor. Thus, LBD require grafts to promote defect repair and bone regeneration.

To date, autograft, allograft or bone engineering material implantation for LBDs still has relatively poor satisfaction^3^. In recent years, researchers proposed the application of MSCs in bone defect repair^22^. MSCs were firstly discovered and separated from bone marrow tissues, which had the capacities to differentiate into various cell lineages, such as osteoblasts, chondrocytes and adipocytes^23^. However, bone marrow aspiration always causes pain at the puncture point and source of bone marrow for BM-MSCs isolation is limited. In contrast, muscle tissues accounting for approximate 35% of body weight ensures the source for obtaining M-MSCs, and additionally, M-MSCs are easy to be isolated and cultured *in vitro*. Yang *et al*. made a comparison on the osteogenesis capacity between M-MSCs and BM-MSCs, and found that M-MSCs had stronger proliferation, calcification and osteogenesis capacities than BM-MSCs^24^. Therefore, M-MSCs can completely replace the role of BM-MSCs in the osteogenesis process and serve as one of the ideal seed cells in bone engineering. Note that muscle-derived stem cells have two distinct populations, muscle satellite cells and M-MSCs^25^. In our study, the cell type of muscle-derived stem cells was determined via biomarkers. Results displayed that more than 80% cells were positive for M-MSCs markers CD90 and CD105, and negative for blood cell marker CD45. It proved that the stem cells in this study were not blood cells or muscle satellite cells but M-MSCs.

Although M-MSCs have been reported to exhibit marvelous therapeutic effect in the repair of muscle, nerve, cartilage, and other tissues^26^, M-MSCs monotherapy in this study failed to repair LBD even after 90 d post-operation. HE staining results showed that M-MSCs group had significant larger repaired area as compared with blank control group, but it failed to achieve the morphological integrity. Additionally, tetracycline-labeled new bone areas and new mineralized bone area labeled by Gomori staining were both less than 50% of the total defect area, and Lane-Sandhu histological score of M-MSCs group was 6.5. Micro-CT results showed that compared with blank control group, cancellous bones begun to remodel in M-MSCs group, but the defects were still not completely closed. It indicates that after M-MSCs monotherapy, the defect repair was improved, but the repair effect was not ideal. The application of M-MSCs alone in repairing LBD was unsatisfactory. Thus, combination therapy of cytokine and M-MSCs was required.

PRP is an autologous biologic derived from centrifugated autologous plasma. It is enriched not only by a high level of platelets, but also by a range of leukocytes, growth factors, chemokines, cytokines, and other plasma proteins. The proportion of various cytokines in PRP is also the same as that in the body, and PRP was reported to promote the repair of bone and cartilage damages^27^. However, in our study, the repair capacity of PRP monotherapy for LBDs was also limited. At 90 d after defect construction, morphological analysis, Lane-Sandhu histological evaluation and Micro-CT results showed that compared with blank control, PRP monotherapy could promote bone defect repair, but its repair effect was much poorer than single treatment of M-MSCs. It suggests PRP monotherapy exhibited limited repair capacity in LBD repair.

However, in M-MSCs/PRP group, HE staining showed that after 90 d of treatment, more than 80% of defect area were repaired, and much more new bone tissues regenerated. The area of tetracycline-labeled new bone tissues and new mineralized bone are in M-MSCs/PRP group were elevated significantly as compared with blank control group or M-MSCs group. In addition, Lane-Sandhu histological score reached 9, suggesting that the bone repair was basically completed. Micro-CT results displayed that in M-MSCs/PRP group, after 90 d of treatment, cancellous bones were completely remodeled and bone defects was completely closed, which was defined as imageological recovery. BMD was also significantly improved in M-MSCs/PRP group as compared with blank control group or M-MSCs group. These results indicate that M-MSCs/PRP combined therapy presented better regeneration capacity compared with M-MSCs monotherapy, and it could promote the recovery of LBD within 90 d. The application of PRP conferred significant enhancement on the osteogenic capacity of M-MSCs. Additionally, this study is the first to demonstrate the application value of M-MSCs/PRP combined therapy in the LBD repair through rabbit model.

Further experiments also revealed the molecular mechanism of PRP in enhancing the regeneration capacity of M-MSCs. PRP treatment induced the migration, proliferation of M-MSCs. It also promoted the osteogenic differentiation while inhibited muscular differentiation of M-MSCs. These effects of PRP treatment may derive from its contained growth factor and chemotactic factor, such as TGF-β1, VEGF, IGF-1, which has been reported to induce the migration, proliferation and osteogenic differentiation of MSC *in vitro*^28^. However, the specific regulatory network of PRP still need further explorations. Additionally, our result is also consistent with Katrina’s study^29^. He highlights that using platelet products to enhance the migration and proliferation of MSCs is a better approach for bone regeneration^30^. As cell migration of MSCs from the edge of defect and constantly proliferation to generate new cells are important for regeneration, the in vitro molecular mechanisms of PRP may underlie the strong regeneration capacity of M-MSCs/PRP combined therapy.

## Conclusion

This study provided experimental evidence that in LBD rabbit model, M-MSCs/PRP combined therapy exhibited significant improvement in morphological and histological bone repair. After 90 d post-operation, M-MSCs/PRP combined therapy also promote the imageological recovery of bone defects, which was much better than natural bone repair, M-MSCs monotherapy or PRP monotherapy. The molecular mechanism of PRP application in enhancing the regeneration capacity of M-MSCs was manifested by promoting the migration and growth of M-MSCs into defect area while inducing the osteogenic differentiation. This is the first study to demonstrate the application significance of M-MSCs/PRP combined therapy in LBD and the crucial molecular mechanism of PRP treatment. It provided a basis for the clinical application and popularization of M-MSCs/PRP combined therapy. However, further exploration on sex^31^ or age^32^ factor in the combination therapy in LBD is required.

## Supplementary information

Supplementary information .
