# Supplementary material for: Platelet-rich plasma enhances the repair capacity of muscle-derived mesenchymal stem cells to large humeral bone defect in rabbits
Source: Sci Rep. 2020 Apr 21;10:6771. doi: 10.1038/s41598-020-63496-5 (PMC7174361; doi:10.1038/s41598-020-63496-5)
Supplement: Supplementary file 1 — Supplementary information . [file 41598_2020_63496_MOESM1_ESM.pdf]

Supplementary Information

Title Page

Platelet-rich plasma enhances the repair capacity of muscle-derived mesenchymal stem cells to large humeral bone defect in rabbits

Nuo Yin[#], Yifei Wang[#], Liang Ding, Junjie Yuan, Li Du, Zhongsheng Zhu, Mingmang Pan, Feng Xue[*], Haijun Xiao[*]

Department of orthopedics, Shanghai Fengxian District Central Hospital, Shanghai, China, 201499.

[#]These authors contributed equally: Nuo Yin and Yifei Wang.

[*]Corresponding authors:

Dr. Feng Xue
Affiliation: Department of orthopedics, Shanghai Fengxian District Central Hospital
Address: No. 6600 Nanfeng Highway, Fengxian District, Shanghai
E-mail: xuemd@yahoo.com; Tel: 57424074

Dr. Haijun Xiao
Affiliation: Department of orthopedics, Shanghai Fengxian District Central Hospital
Address: No. 6600 Nanfeng Highway, Fengxian District, Shanghai
E-mail: xiaohaijun89@126.com; Tel: 57424074

Items		Socre
Bone connection	No connections	0
	Fibrous connections	1
	Connections of bone and bone-like tissue	2
	Connections of bones	3
	Complete bone regeneration	4
Cancellous bone	No activity of osteocytes	0
	Early aggregation of new bone	1
	Activity of new bone aggregation	2
	Cancellous bone is remoulding	3
	Cancellous bone remoulds completely	4
Cortical bone	No growth of cortical bone	0
	Early growth of cortical bone	1
	Cortical bone is remoulding	2
	Most Cortical bone completes remoulding	3
	Cortical bone remoulds completely	4

Supplementary Table 1. Lane-Sandhu histological evaluation scale

For gels/blots quality check

1. Full length picture of original gels/blots (low-contrast)

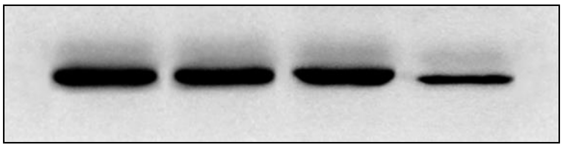

MyoD1

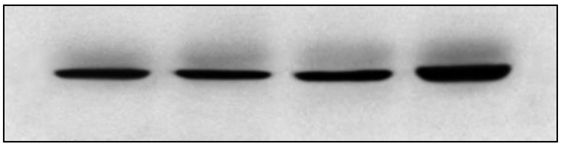

Cbfa-1

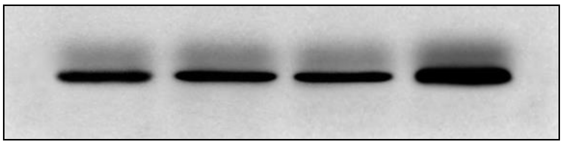

Coll I

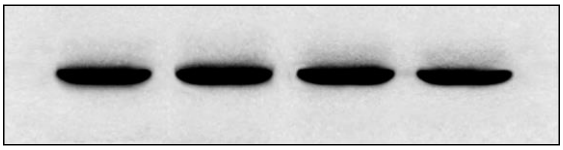

β-actin

2. Combination and cropping

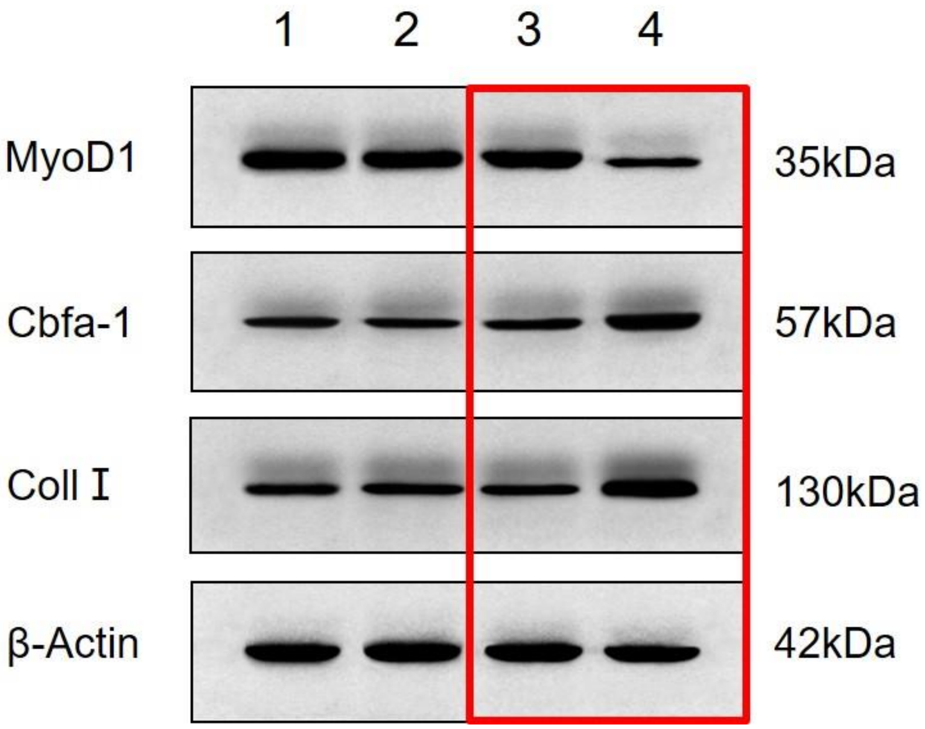

Group information:

1. M-MSCs treated with 1% FBS (Hyclone)
2. M-MSCs treated with 10% FBS (Gibco)
3. M-MSCs treated with 10% FBS (Hyclone)
4. M-MSCs treated with PRP.

Group 1. 2. 3 were set to explore the effects of different concentration of FBS or FBS

from different brand on the differentiation of M-MSCs. Due to no significant differences were observed, this part was deleted. The red border stands for the cutline of cropping.
